# Development of live attenuated Enterovirus 71 vaccine strains that confer protection against lethal challenge in mice

**DOI:** 10.1038/s41598-019-41285-z

**Published:** 2019-03-18

**Authors:** Pinn Tsin Isabel Yee, Soon Hao Tan, Kien Chai Ong, Kuan Onn Tan, Kum Thong Wong, Sharifah Syed Hassan, Chit Laa Poh

**Affiliations:** 1 Centre for Virus and Vaccine Research, School of Science and Technology, Sunway University, Bandar Sunway, Selangor 47500 Malaysia; 2 Department of Biomedical Science, Faculty of Medicine, University of Malaya, Jalan University, 50603 Kuala Lumpur, Selangor Malaysia; 3 Department of Biological Sciences, School of Science and Technology, Sunway University, Kuala Lumpur, Selangor 47500 Malaysia; 4 Department of Pathology, Faculty of Medicine, University of Malaya, Jalan University, 50603 Kuala Lumpur, Selangor Malaysia; 5 Jeffrey Cheah School of Medicine and Health Sciences, Monash University, Jalan Lagoon Selatan, Bandar Sunway, 47500 Subang Jaya, Malaysia

## Abstract

Besides causing mild hand, foot and mouth infections, Enterovirus A71 (EV-A71) is associated with neurological complications and fatality. With concerns about rising EV-A71 virulence, there is an urgency for more effective vaccines. The live attenuated vaccine (LAV) is a more valuable vaccine as it can elicit both humoral and cellular immune responses. A miRNA-based vaccine strain (pIY) carrying let-7a and miR-124a target genes in the EV-A71 genome which has a partial deletion in the 5′NTR (∆11 bp) and G64R mutation (3D^p^°^l^) was designed. The viral RNA copy number and viral titers of the pIY strain were significantly lower in SHSY-5Y cells that expressed both let-7a and miR-124a. Inhibition of the cognate miRNAs expressed in RD and SHSY-5Y cells demonstrated de-repression of viral mRNA translation. A previously constructed multiply mutated strain, MMS and the pIY vaccine strain were assessed in their ability to protect 4-week old mice from hind limb paralysis. The MMS showed higher amounts of IFN-γ *ex vivo* than the pIY vaccine strain. There was absence of EV-A71 antigen in the skeletal muscles and spinal cord micrographs of mice vaccinated with the MMS and pIY strains. The MMS and pIY strains are promising LAV candidates developed against severe EV-A71 infections.

## Introduction

The hand, foot, and mouth disease (HFMD) is generally manifested as a mild illness but neurological complications such as encephalomyelitis, acute flaccid paralysis and aseptic meningitis have occurred in infants and young children below 6 years of age^1^. Enteroviruses such as Enterovirus 71 (EV-A71), Coxsackievirus type A16 (CV-A16) and other enteroviruses causing HFMD have led to over 7 million infections, including 2457 fatalities in China from 2008 to 2012^2^. Most HFMD infections that led to fatality were due to the EV-A71 virus^3^. Since 1997, countries such as Taiwan, Malaysia, Singapore and Vietnam have experienced cyclical epidemics which occurred every 2 or 3 years. EV-A71 was first isolated as the etiological agent of HFMD from a young child in California, United States in 1969^4^. The virus is a member of the human Enterovirus Species A group within the family *Picornaviridae*^5^.

With rising concern about the virulence of EV-A71, there is an urgent need for a vaccine against EV-A71 to be produced that is approved by FDA. China’s FDA has issued drug certificates and production licenses for the inactivated vaccine (IV) from 3 companies namely, Sinovac, Vigoo and CAMS^6^. An IV was found to elicit weak T cell responses because there were decreased amounts of antigen in the IV to sustain prolonged antibody responses^7^. The efficacy of the IVs against EV-A71 is predominantly dependent on antibody-mediated protection. The surface proteins of viruses will continuously undergo genetic changes through evolution of the genome and this could affect effective neutralization of newly evolved strains by antibodies elicited by the current vaccine strain^8^. Since IVs do not elicit long-term immune memory, it would also require several booster vaccinations after administration. The live attenuated vaccine (LAV) may serve as a more effective vaccine as it can elicit *both* humoral and cellular immune responses. Recent studies indicated that cellular and not humoral immunity determines the clinical outcome of EV-A71 infections as there was no difference in NtAb titers between mild and fatal HFMD cases^9^. It was discovered that approximately 93% of T cell responses were induced by antigens from the structural VP2 region as compared with antigens from VP1, VP3 and VP4 after *in vitro* expansion. These cellular responses were predominantly from the IFN-γ-CD4^+^ T cells and not from the CD8^+^ T cells^10^.

MicroRNAs (miRNAs) are 20–24 nucleotides long, non-coding RNAs that can prevent translation of messenger RNA (mRNA)^11^. Insertion of miRNA into a viral genome has been shown to control viral tropism. The virus expressing the corresponding miRNA would not be able to replicate in cells that carried the specific miRNA and would thereby display an attenuated phenotype^12^. The human genome has more than 1000 different miRNAs that are tissue specific and can function as post-transcriptional regulators, capable of repressing hundreds of genes and regulating many cellular processes^13,14^. An ideal attenuated virus vaccine should be one that does not replicate in tissues to cause disease and yet at the same time, replicate sufficiently in other tissues to elicit a long-lasting immune reaction^15^. Using the miRNA-based approach, Barnes *et al*.^12^ inserted miRNA coding sites for let-7a or miR-124a into the poliovirus genome and showed that there was restricted viral replication in the murine central nervous system (CNS). Although the degree of attenuation of neuro-virulence exceeded 5 orders of magnitude, these engineered miRNA viruses were still able to replicate in non-neuronal tissues and elicited high neutralizing antibody (NtAb) titers. Thus, the viruses encoding miRNAs could stimulate a strong protective immunity without the adverse risk of causing paralysis in the host.

Previously, we had identified significant genetic determinants of virulence in the EV-A71 virus (sub-genotype B4 virus; 5865/Sin/000009) and the information was used to construct the multiply mutated EV-A71 strain, MMS which carried a substitution of nucleotide U to C (U700C) at the 5′-NTR, replacement of amino acid (aa) E to G at VP1-145 (E145G), 3 amino acid replacements at positions 98, 242, and 244 of VP1 and replacement of an amino acid at position 64 of the 3D^p^°^l^ (G64R) in the mutant that had a partial deletion (∆11 bp in 5′-NTR)^16^. In this study, we constructed the EV-A71 miRNA-based vaccine strain (pIY) to carry let-7a and miR-124a target genes. As cellular miRNAs have such diverse tissue-specific distributions, the attenuation effects of the pIY vaccine strain carrying both miRNAs let-7a and miR-124a were assessed to demonstrate that the vaccine strain could indeed decrease viral pathogenesis in different cell types. This attenuation could be due to the binding between the endogenous cellular miRNA and the viral miRNA target sequences, inducing miRNA cleavage and causing significant reduction of viral replications. We evaluated the ability of the MMS and the miRNA strain (pIY) to act as vaccines in protecting mice against a mouse adapted virus strain (MAV) in challenge studies^17^.

## Results

### Construction of the recombinant pIY vaccine strain carrying microRNAs

**Figure 1:**
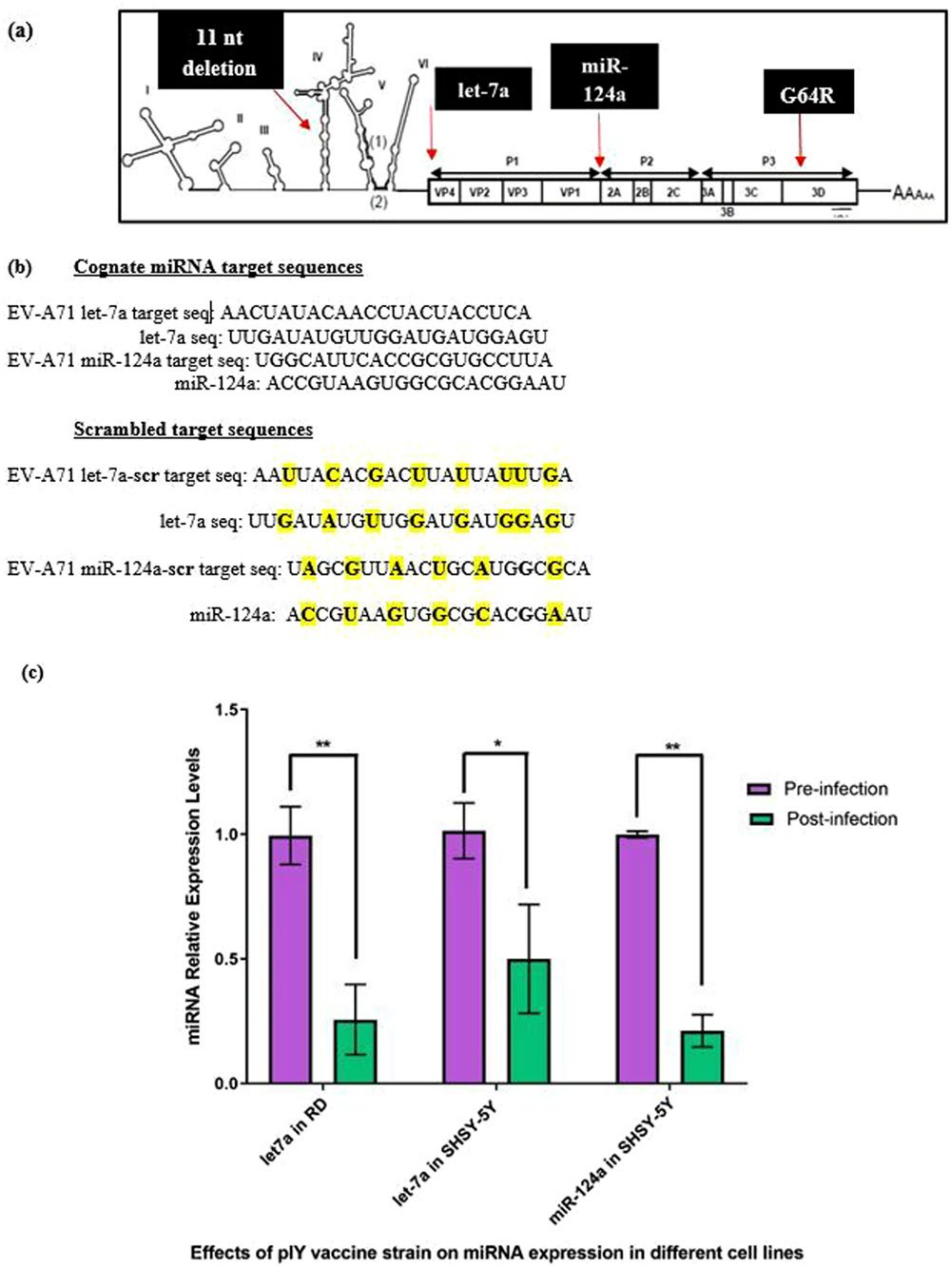
Engineering EV-A71 virus sub-genotype B4 strain 41 to carry 2 miRNA target sequences and miRNA relative expression levels of let-7a and miR-124a in cell lines. (**a**) Target sequences complementary to the two miRNAs (let-7a and miR124a) were inserted into 2 locations within the EV-A71 genome. The miRNA target sequence for let-7a was inserted at the 3′end of the 5′-NTR. The target sequence for miR-124a was located between the VP1 structural and 2A non-structural region. (**b**) Perfect sequence complementarity between the target sequence and miRNA inserted into the EV-A71 genome. Scrambled target sequences referred to imperfect complementarity between the scrambled target sequence and miRNA inserted into the EV-A71 genome. (**c**) Relative expression levels of let-7a and miR-124a in RD and SHSY-5Y cells after pIY vaccine strain pre-infection and 24 h post-infection. The relative expression was calculated with reference to control samples comprising cognate endogenous miRNA levels in the respective cell lines before pIY infection. Error bars indicate the standard deviation of the mean; P-values between the different groups with the one-way ANOVA. *P < 0.05, **P < 0.01.

Two microRNA target genes, let-7a and miR-124a, were inserted into the EV-A71 mutant strain that carried a partial deletion (nt. 475–485 in the 5′-NTR) and a substitution of nucleotide at position 6129 (nt G to nt C) at the 3D^p^°^l^ region (G64R). The miRNA target sequence for let-7a was inserted at the 3′end of the 5′-NTR in the EV-A71 genome while the target sequence for miR-124a was located between the structural (VP1) and non-structural (2 A) genes within the coding region (Fig. 1a). Recombinant EV-A71 genomes carrying the miRNA let-7a, miR-124a target genes and recombinant EV-A71 genomes containing scrambled miRNA genes were generated. The recombinant plasmid containing the correct target sequences (pTOPO-miRNA7124) and recombinant plasmid carrying scrambled sequences were confirmed by sequencing (Fig. 1b). The recombinant plasmid pTOPO-miRNA7124 that carried the let-7a and miR-124a target genes, and the G64R mutation in the EV-A71 genome with 11 bp deletion in the 5′-NTR was transformed into a competent *E*.*coli* strain. After *in vitro* transcription, infectious viral RNAs that were produced were transfected into RD cells with Lipofectamine 2000. The recombinant viruses released from RD cells upon lysis was designated as the pIY miRNA vaccine strain.

Quantification of the levels of the viral RNA copy number in RD and SHSY-5Y cells before and after infection (24 h) with the pIY strain was determined. There was a significant 3-fold decrease (P = 0.0082) in let-7a miRNA relative expression levels in RD cells when comparing pre- and post-infection (24 h) with the pIY strain. This is expected as the cognate let-7a in RD cells would bind to the complementary let-7a target sequence in the pIY strain and thereby, reduce the expression levels of let-7a. For SHSY-5Y cells, the let-7a expression levels (P = 0.0012) decreased after 24 h post-infection with the pIY vaccine strain. There was also a significant 4-fold reduction (P = 0.0064) in miR-124a expression levels when comparing the pre- and post-infection stages of pIY strain in SHSY-5Y cells (24 h). This could be attributed to the abundance of neuronal miR-124a in SHSY-5Y cells (Fig. 1c).

### Quantitation of viral titer in different cell lines

**Figure 2:**
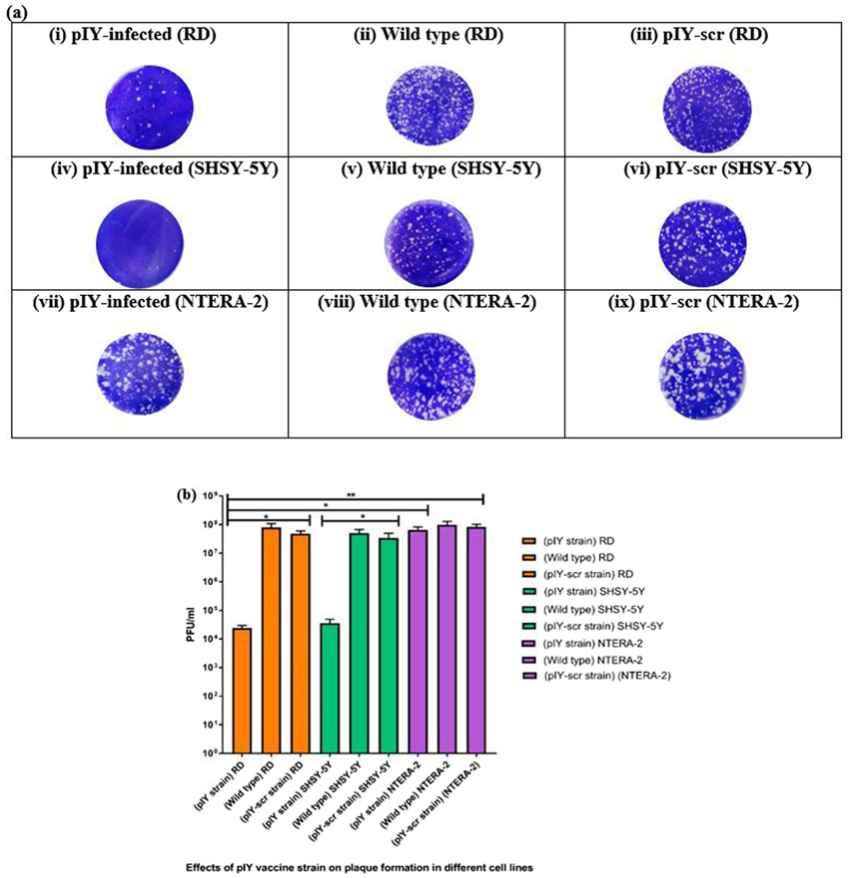
Quantification of viral titer (PFU/ml) in different cell lines. (**a**) The plaque assays were performed in monolayer cells. PFU/ml caused by (i) pIY-infected RD cells (ii) WT EV-A71 strain 41 in RD cells (iii) pIY-scr-infected RD cells (iv) pIY-infected SHSY-5Y cells (v) WT EV-A71 strain 41 in SHSY-5Y cells (vi) pIY-scr-infected SHSY-5Y cells (vii) pIY-infected NTERA-2 cells (viii) WT EV-A71 strain 41 in NTERA-2 cells (ix) pIY-scr-infected NTERA-2 cells. (**b**) PFU/ml of the pIY vaccine strain, WT EV-A71 strain 41 and pIY-scr strain in three different cell lines. PFU/ml are the average of three biological replicates. Error bars indicate the standard deviation of the mean; P-values between the different groups with the one-way ANOVA. *P < 0.05, **P < 0.01.

Replication of the pIY miRNA vaccine strain carrying the two microRNAs in RD, SHYS-5Y and NTERA-2 cells were evaluated by plaque assays at a MOI of 0.1. The RD cells infected with the pIY strain exhibited significant reduction of viral titer to 2.4 × 10^4^ PFU/ml in RD cells (P = 0.0443) (Fig. 2ai). The EV-A71 wild-type (WT) virus infection in RD cells led to as high as 8.0 × 10^7^ PFU/ml (Fig. 2aii). As expected, the recombinant virus carrying the pIY scrambled sequences (pIY-scr) in infected RD cells generated viruses with a PFU/ml of 4.8 × 10^7^, this was similar to that observed for the wild type EV-A71 (Fig. 2aiii). The SHSY-5Y cells infected with the pIY strain exhibited significant reduction of viral titer to 3.5 × 10^4^ PFU/ml in SHSY-5Y cells (P = 0.0325) (Fig. 2aiv). The EV-A71 WT virus infection in SHSY-5Y cells reached as high as 5.0 × 10^7^ PFU/ml in SH-SY5Y cells (Fig. 2av). The virus carrying the pIY scrambled sequences (pIY-scr) in infected SH-SY5Y cells generated viruses with a viral titer of 3.4 × 10^7^ PFU/ml (Fig. 2avi).

However, infection by recombinant pIY in NTERA-2 cells exhibited no significant (P = 0.5029) reduction of viral titer, with a viral count of 6.5 × 10^7^ PFU/ml (Fig. 2avii). This is similar to that observed for the WT virus which generated 9.8 × 10^7^ PFU/ml in NTERA-2 cells (Fig. 2aviii). The pIY-scr-infected NTERA-2 cells had viral titer of 8.2 × 10^7^ PFU/ml, a value which is similar to the viral titer achieved by infection of the wild type EV-A71 (Fig. 2aix). As NTERA-2 cells did not express endogenous let-7a and miR-124a, there was no cognate miRNAs binding to the complementary target genes on the pIY vaccine strain to reduce miRNA expression and subsequent generation of infectious viral particles^12^. There was a significant 3-log reduction of viral titer (P = 0.0325) when the PFU/ml values were compared between the pIY-infected SHYSY-5Y cells (3.6 × 10^4^ log_10_ PFU/ml) and the EV-A71 wild type-infected SHYSY-5Y cells (5.0 × 10^7^ log_10_ PFU/ml) (Fig. 2b). As there was reduction in viral growth, this is indicative that the plaque forming capability of the pIY vaccine strain was significantly reduced in both RD and SHSY-5Y cells due to the presence of their respective cognate miRNAs. This signifies that there would be weaker viremia due to a reduction in viral copy number as higher viral load is known to correlate with higher virulence.

### Viral RNA copy determination in different cell lines

**Figure 3:**
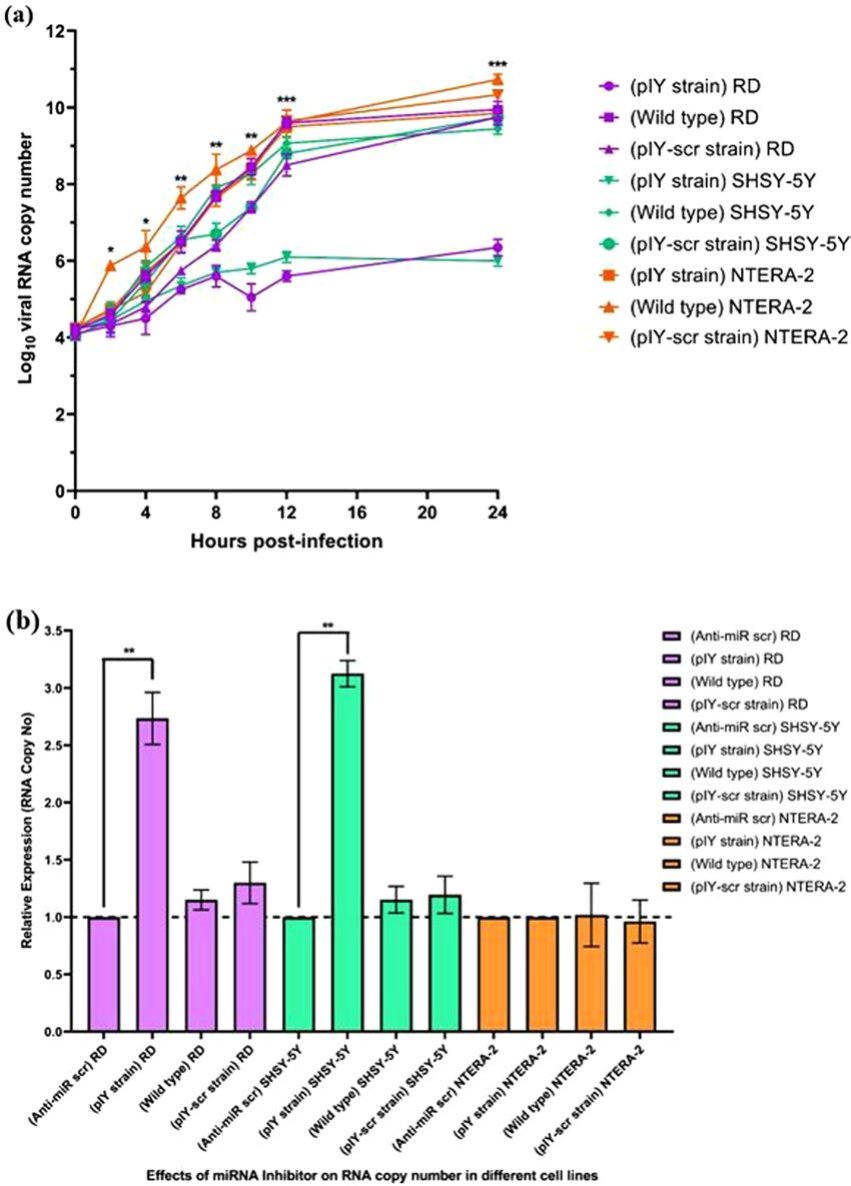
Quantitation of viral RNA copy number in RD, SHSY-5Y and NTERA-2 cells. (**a**) Growth kinetics study of the pIY vaccine strain in RD, SHSY-5Y and NTERA-2 cells. Statistical analysis was done across all infected groups using a Mann–Whitney *U*-test. (**b**) Inhibition of the expressions of endogenous let-7a and miR-124a using anti-miR oligonucleotides corresponding to the let-7a and miR124a complementary sequence. Normalized RNA copy numbers are shown as fold differences of the scrambled anti-miR oligonucleotide control. Different cell lines were transfected with 25 pmol of anti-miR for let-7a and miR-124a and were then infected with pIY strain, pIY-scr and wild type EV-A71. The viral RNA copy numbers were quantified after 0, 2, 4, 6, 8, 10, 12, and 24 h post-infection by Real-Time TaqMan RT-PCR and is the average of three biological replicates. Error bars indicate the standard deviation of mean; P-values between the varying groups with the one-way ANOVA. *P < 0.05, **P < 0.01, ***P < 0.001.

When overnight grown RD/SHSY-5Y/NTERA-2 cells (1.5 × 10^5^ cells/well in a 24-well plate) were infected with the pIY vaccine strain, pIY-scr and WT, the growth kinetics was measured after 0, 2, 4, 6, 8, 10, 12, and 24 h post-infection. The pIY strain from infected NTERA-2 cells yielded significantly higher RNA copy number than the pIY strain from infected SHYSY-5Y or RD cells at all hpi. By 24 hpi, the pIY strain from infected SHYSY-5Y cells had 1.7-log_10_ lesser RNA copy number (5.8 ± 0.2 log_10_ RNA copies/ml), as compared to the pIY recovered from infected NTERA-2 cells (9.9 ± 0.1 log_10_ RNA copies/ml) (P = 0.0042). Similarly, the RNA copy number of pIY recovered from RD cells (6.0 ± 0.2 log_10_ RNA copies/ml) was 1.5-log_10_ lesser than the pIY recovered from infected NTERA-2 cells (9.9 ± 0.1 log_10_ RNA copies/ml) (P = 0.0072) (Fig. 3a).

By 24-hour post infection, the difference between viral RNA copy numbers of NTERA-2 cells when compared with RD and SHSY-5Y cells was significant, showing attenuation due to presence of let-7a in RD cells and *both* let-7a and miR-124a in SHSY-5Y cells. The pIY strain following infection in RD cells produced 6.0 ± 0.2 log_10_ RNA copies/ml and this is a significant 2-log decrease (P = 0.0003) in RNA copy number than that produced by the WT positive control (10.0 ± 0.2 log_10_ RNA copies/ml). Similarly, 2-log decrease of RNA copy number (5.8 ± 0.2 log_10_ RNA copies/ml) (P = 0.001), was observed in pIY-infected SHSY-5Y cells, when compared to the WT infected SHSY-5Y cells (9.2 ± 0.1 log_10_ RNA copies/ml). The pIY-scr strain produced 9.5 ± 0.2 log_10_ RNA copies/ml when RD cells were infected with pIY-scr strain and the copy number was close to that observed for the WT infected RD cells (10.0 ± 0.2 log_10_ RNA copies/ml). This was also observed for the pIY-scr infected SHSY-5Y cells that produced similar amount of viral RNA copy number (9.7 ± 0.4 log_10_ RNA copies/ml) as that of the pIY-scr infected RD cells. Whereas for the NTERA-2 cells that did not express either of the miRNAs, the log_10_ viral copy number of the pIY strain remained high at a level which was quite comparable to the viral copies produced by the pIY-scr strain infected NTERA-2 cells (10.3 ± 0.1 log_10_ RNA copies/ml) (P = 0.4182) and the WT (10.7 ± 0.1 log_10_ RNA copies/ml) (Fig. 3b). In contrast, the negative control cell cultures without virus infection showed an absence of RNA copy number.

To confirm that the reduction in RNA copy number of the pIY strain in RD and SHSY-5Y cells were due to the presence of the miRNA target genes incorporated in the genome, the expression of the pIY strain in the presence of the respective chemically modified antimiR oligonucleotides were evaluated. With the addition of inhibitors to both let-7a and miR124a, this would cause de-repression of the specific targeted miRNA in the viral genome. Non-infected cells did not express any viral RNA copy number and hence, was not reflected in Fig. 3b. All RNA copy numbers were normalized and shown as fold differences of cells transfected with the scrambled anti-miR oligonucleotide control. Transfection of antimiR-124a and antimiR-let7a RNA oligonucleotides in SHSY-5Y cells dramatically knocked down endogenous miR-124a and let-7a expression and resulted in a significant increase in expression of RNA to 3.1-fold (Fig. 3b).

The RNA expression of the WT (2.7-fold) and the pIY-scr transfected RD cells (1.3-fold) were almost similar to cells transfected with scrambled anti-miR oligonucleotides (Fig. 3b). Similarly, the addition of the two scrambled inhibitors to SHSY-5Y cells produced almost similar RNA expression to that produced by the WT positive control and the pIY-scr transfected SHSY-5Y cells (1.2-fold). In the presence of inhibitors (anti-miR scr), there was no significant difference (P > 0.001) between the RNA expression of the pIY strain and the WT (1.2-fold) when NTERA-2 cells were transfected (Fig. 3b).

### Quantitation of viral infectivity by tissue culture infectious dose (TCID_50_/ml)

**Figure 4:**
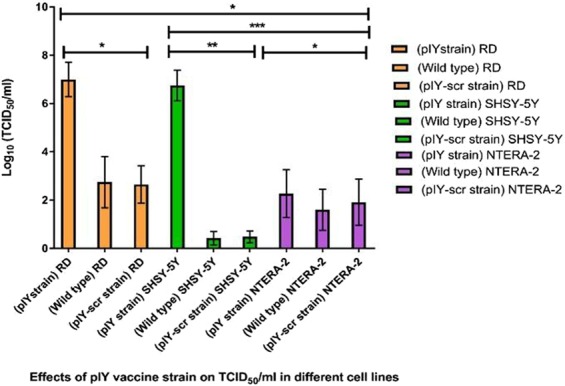
Effects of pIY vaccine strain on TCID_50_/ml in different cell lines. The TCID_50_/ml values were calculated using the Reed and Muench formula determined from at least three independent experiments. Error bars indicate the standard deviation of mean; P-values between the varying groups with the one-way ANOVA. *P < 0.05, **P < 0.01, ***P < 0.001.

Table 1 shows the TCID_50_/ml values obtained from the effects of the pIY vaccine strain in different cell lines. The pIY miRNA vaccine strain in RD cells registered a significant (P = 0.0239) 4-log increase of log_10_ TCID_50_/ml (7.0 ± 0.5), when compared to the WT EV-A71 control (2.7 ± 0.8 log_10_ TCID_50_/ml). This observation is expected as the RD cells expressed cognate let-7a (Fig. 4). The miRNA vaccine strain (pIY)-infected SHSY-5Y cells displayed 5-log increase of 6.7 ± 0.5 log_10_ TCID_50_/ml, in comparison to the WT EV-A71 strain (0.5 ± 0.4 log_10_) (P = 0.0015). This could be explained by the presence of both let-7a or miR-124a in SHSY-5Y cells. This indicated that the pIY vaccine strain would need a significantly higher amount of virus to elicit a cytopathic effect (CPE) in 50% of the of the infected SHSY-5Y cells.

**Table 1:** Tissue culture infectious dose (TCID_50_/ml) of the recombinant pIY vaccine strain^a^.

Recombinant pIY vaccine strain in different cell lines	TCID_50_/ml
pIY- infected RD cells	1.00 × 10^7^
EV-A71 strain 41 in RD cells	5.60 × 10^2^
pIY-scr-infected RD cells	4.47 × 10^2^
pIY- infected SHSY-5Y cells	5.60 × 10^6^
EV-A71 strain 41 in SHSY-5Y cells	0.30 × 10
pIY-scr-infected in SHSY-5Y cells	0.50 × 10
pIY-transfected NTERA-2 cells	1.85 × 10^2^
EV-A71 strain 41 in NTERA-2 cells	5.61 × 10
pIY-scr-infected NTERA-2 cells	9.30 × 10

^a^The recombinant pIY vaccine strain carried 2 miRNA genes, as well as mutations in the 5′-NTR (∆11 bp) and G64R in the 3D polymerase.

In addition, the pIY strain in NTERA-2 cells (2.3 ± 0.6 log_10_ TCID_50_/ml) showed no significant difference (P = 0.7444) to the WT strain 41 which expressed 1.7 ± 0.5 log_10_ TCID_50_/ml and the pIY-scr strain in NTERA-2 cells (2.0 ± 0.6 log_10_ TCID_50_/ml). This observation could be explained by the lack of both miRNAs, let-7a and miR-124a, in NTERA-2 cells. Hence, there was no inhibition of EV-A71 mRNA translation in NTERA-2 cells. The pIY-infected SHSY-5Y and RD cells required 5-log increase of viruses to elicit CPE in 50% of the cells, when compared with the pIY-scr strain in SHSY-5Y which expressed 0.7 ± 0.2 log_10_ TCID_50_/ml (P = 0.0058) and 2.7 ± 0.6 log_10_ in RD cells (P = 0.0280), respectively. The TCID_50_/ml of the pIY-scr viral strain in RD cells also displayed similar value (2.7 ± 0.8 log_10_ TCID_50_/ml) to the WT strain 41 (RD cells). The results indicated a high degree of attenuation of the pIY vaccine strain carrying the 2 miRNA target sequences in both SHSY-5Y and RD cells.

### Genetic stability of the pIY miRNA vaccine strain in comparison to the MMS

Since both the multiply mutated strain, MMS and the pIY miRNA vaccine strains have shown significant reduction in virulence, it is necessary to assess the genetic stability of each of the mutations present in the viral genome by examining the potential of reversion of both types of mutants. This was performed by serial passaging and recovery of the viral strain after a series of 20 passages in RD cells. In the pIY miRNA vaccine strain, both miRNA target sequences viz. let-7a (AACTATACAACCTACTACCTCA) and miR-124a (TGGCATTCACCGCGTGCCTTA) were stable after 20 passages in RD cells. Studies performed to assess the genetic stability of the MMS showed that each of the 6 single site mutations and the deletion mutation were stable in the MMS and did not revert to the WT genotype after 20 passages. The mutations that were stable in the MMS vaccine strain were present at the 5′-NTR (U700C), E145G at VP1–145, amino acid replacements at positions 98, 242, and 244 of VP1, G64R in the 3D^p^°^l^ and the partial deletion (11 bp) in the 5′-NTR. The significant reduction in virulence of the virus carrying miRNAs let-7a and miR-124a target sequences in tissue culture cells have led us to further evaluate the effect of the miRNA vaccine strain on viral pathogenesis in 4-week old ICR mice.

### Protective efficacy of the pIY miRNA vaccine strain in comparison to the MMS

Vaccination using the EV-A71 pIY miRNA and the MMS vaccine strains to prevent severe EV-A71 infection was investigated, whereby 4-week old mice were challenged with 10^5^ CCID_50_/ml of the mouse-adapted virus (MAV) strain that was originally isolated from a fatal EV-A71 human infection^18^. The inactivated vaccine (IV) strain is the EV-A71 WT sub-genotype B4 strain 41 which was subjected to heating at 56°C for 30 mins. This IV strain was tested for non-viability after infection of RD cells at a MOI of 0.1. As there was absence of cytopathic effect in the RD cells even after 48 h of incubation, the IV was considered sufficiently inactivated and was ready to be used as a vaccine strain.

**Figure 5:**
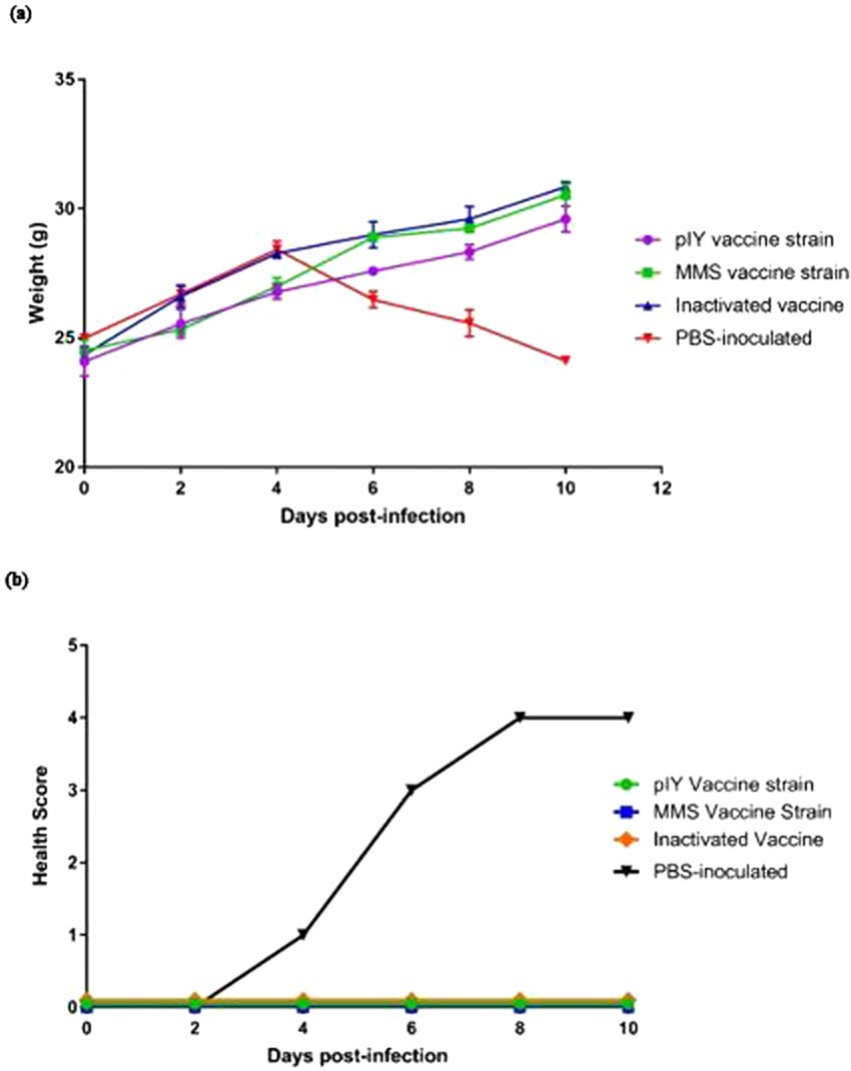
Protective efficacy conferred by vaccine strains in mice from lethal EV-A71 challenge. (**a**) Body weights were recorded for all 4-week old ICR mice after EV-A71 challenge until 10 days’ post-infection. Besides weight gain as an indicator of protective efficacy, other parameters such as alertness, activity and normal appetite were assessed. The results are expressed as the mean ± standard error of the mean of five mice. (**b**) Clinical scores of challenge with the EV-A71 MAV strain in 4-week old ICR mice inoculated with the pIY miRNA vaccine strain, MMS, and the IV. The control group was the mice administered with PBS but challenged with the mouse adapted virus (MAV) (n = 5).

Protection experiments showed that mice inoculated with the pIY miRNA vaccine strain, MMS or IV strain inoculated by the intraperitoneal (i.p.) route were able to confer 100% protection from hind limb paralysis against the EV-A71 MAV challenge. All five mice from the PBS-inoculated group showed significant weight loss and hind limb paralysis on day 5-post infection. In contrast, the pIY miRNA vaccine strain, MMS and IV-inoculated mice showed no signs of weight loss but registered a steady increase in weight after EV-A71 MAV challenge (Fig. 5a). Mice that were inoculated with PBS reached an average health score of 4 at day 8 post-infection. This was based on the health score of clinical disease which was evaluated as follows: 0, healthy; 1, ruffled hair, hunchbacked (day 4); 2, reduced mobility; 3, limb weakness (day 6); and 4, hind limb paralysis (days 8–10). These results showed that when the pIY miRNA vaccine strain and the MMS were administered by the i.p. route, they were able to confer 100% protection against hind limb paralysis following EV-A71 (MAV) challenge (Fig. 5b).

### Production of Interferon-γ in response to stimulation by vaccine strains

**Figure 6:**
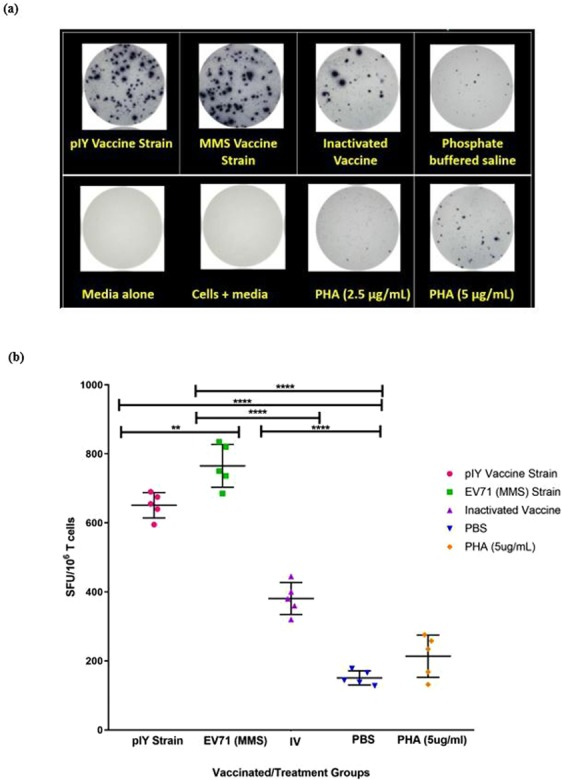
IFN-γ produced by mice inoculated with the pIY vaccine strain, the MMS and the IV. (**a**) The PBS-inoculated mice (n = 5) served as the negative control group. PHA-M (2.5 µg/ml and 5 µg/ml) served as the positive controls for the IFN-γ assay. (**b**) Frequencies of cells producing IFN-γ in response to different vaccine strains measured using IFN-γ ELISPOT. The assays were carried out using vaccine-stimulated mice splenocytes incubated at 37 °C for 24 h. Data are expressed as SFU/10^6^ T cells. ELISPOT positivity was defined as > 50 SFU/10^6^ T cells. Error bars represent median and interquartile range; P-values between groups with the one-way ANOVA. **P < 0.01, ****P < 0.0001.

Mice splenocytes were tested *ex vivo* against different vaccine strains for the production of IFN-γ by the ELISPOT assay. As the assay was performed *ex vivo*, this reflected the closest probable indicator of the human *in vivo* response. The splenocyte cells were stimulated for 24 h in the presence or absence of the pIY vaccine strain, the MMS or the IV. The MMS vaccine strain induced the highest IFN-γ response (765 SFU/10^6^ T cells) *ex vivo*, followed by the pIY vaccine strain (640 SFU/10^6^ T cells) and the IV (400 SFU/10^6^ T cells) (Fig. 6a,b). Low levels of IFN-γ secretion was also detected in the negative control group that received PBS (50 SFU/10^6^ T cells), albeit at a much significantly lower level (P < 0.001) than those elicited by the MMS and pIY vaccine strains. No response was detected in the wells containing media alone or media and cells which served as negative controls. Phytohemagglutinin-M (PHA-M) at 2.5 µg/ml and 5 µg/ml were tested to determine the optimal concentration for stimulation of IFN-γ in splenocytes. The cellular response to 5 µg/ml PHA-M was strong with the samples and was a postive indicator that the ELISPOT assay was functioning at optimal levels.

### Immunohistochemical staining of EV-A71 presence in organs of the vaccinated mice

**Figure 7:**
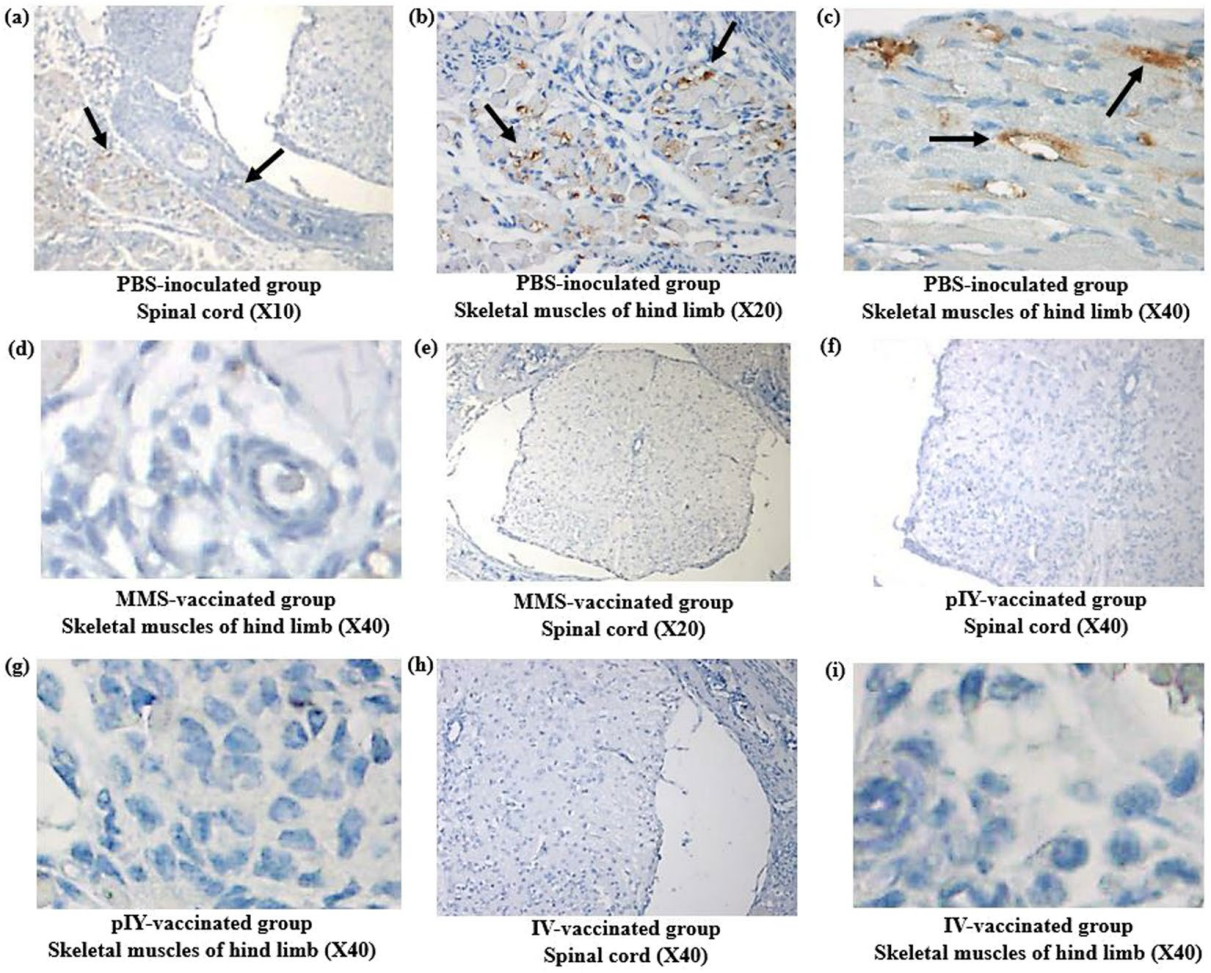
Tissue sections of mice vaccinated with pIY, MMS and IV in comparison with PBS inoculated group. The tissue sections were incubated with murine monoclonal antibody against EV-A71 VP1 as the primary antibody. Then the tissues were incubated with a peroxidase labelled polymer conjugated to goat anti-mouse IgG. Arrows indicate presence of viral antigen in the (**a**) spinal cord and (**b**,**c**) skeletal muscles of PBS-inoculated mice. Representative images indicating absence of EV-A71 viral antigen in the (**d**) skeletal muscles and (**e**) spinal cord of MMS-vaccinated mice, (**f**) spinal cord and (**g**) skeletal muscles of pIY-vaccinated mice, (**h**) spinal cord and (**i**) skeletal muscles of IV-vaccinated mice. Scale bars, 100 µM.

The presence of the pIY vaccine strain, the MMS and the IV in mice was determined by immunohistochemical staining of the skeletal muscles and spinal cords harvested on day 10-post infection. For the PBS-inoculated group, the EV-A71 viral antigen was detected in the spinal cord and skeletal muscles of the hind limbs using immuno-histochemical analysis (Fig. 7a–c). In contrast, there was absence of viral antigen in at least 10 micrographs of skeletal muscles of the hind limbs for the MMS (Fig. 7d), pIY (Fig. 7g) and IV-vaccinated groups of mice (Fig. 7i). In the 3 groups of vaccinated mice, there was no sign of inflammation or marked lesions in the tissue cryosections of the spinal cords (Fig. 7e, f and h). The results demonstrated the significance of the MMS, pIY vaccine strains and the IV in their capability to prevent systemic spread and EV-A71 replication *in vivo*.

## Discussion

Enteroviruses such as EV-A71, CV-A6, CV-A8, CV-A10 and CV-A16 have led to over 7 million infections, including 2457 fatalities in China from 2008 to 2012. Five years of epidemiological surveillance in China (2008–2014) showed that 43.73% of HFMD cases were due to EV-A71^19^. Some of these young children died of complications due to pulmonary edema, while others could not survive brain inflammations due to virulent genotypes of EV-A71. The lack of FDA-approved vaccines against EV-A71 highlights the urgency and significance of developing new vaccines against EV-A71 to prevent further fatalities. Pharmaceutical companies such as CAMS, Vigoo and Sinovac have obtained drug certificates and production licenses by China’s FDA for the inactivated vaccine (IV) against sub-genotype C4a^20^. Sinovac reported that the efficacy of their IV against EV-A71-associated hospitalization and HFMD cases with neurologic complications were both 100%^21^. Another study also showed an efficacy rate of 95.1% against EV-A71-associated HFMD for the second year post-vaccination and an overall efficacy rate of 94.7% at two years^22^. It would be interesting to perform phase IV studies to assess the types of immune response and correlates of protection against all EV-A71 genotypes/sub-genotypes. Although the IV induces good humoral immunity, it requires multiple boosters and lacks strong cellular responses required for long-term protection. Therefore, there is a need to develop other types of vaccines which can induce robust humoral and cellular immunity.

The live attenuated vaccine (LAV) could serve as a safe and economical vaccine against HFMD as it can confer live-long immunity. Recent work has shown that it is possible to generate LAVs that have much lower risks of reversion. For example, attenuated high-fidelity mutants of EV-A71 with a single amino acid change, G64R in its 3D Polymerase (3D^Pol^) was shown to significantly reduce viral virulence *in vivo*. The EV-A71 RG/B4-G64R mutant had reduced virulence as it was unable to generate replication-efficient mutations and had markedly lower genetic diversity to withstand a range of selective pressures^23^. Hence, a high-fidelity EV-A71 variant could increase genetic stability of the LAV. Previous success of using LAVs in the prevention of diseases such as poliovirus, yellow fever, rubella, measles, mumps, rabies, influenza and varicella zoster has proven that safe and effective LAVs could be developed through optimization of immunogenicity and genetic stability. One dose of oral administration of OPV could confer lifelong immunity. There are three attenuated strains, Sabin 1, 2 and 3, being used as the OPV. However, the OPV was not safe enough as it was reported that the incidence of Vaccine-Associated Paralytic Poliomyelitis cases (VAPP) for immunocompetent children receiving their first dose of OPV was approximately 1 out of 750, 000 vacinees^24^. This could be due to too few mutations being present or they were genetically unstable. For example, Sabin 2 only carried 2 nt substitutions and this serotype was shown to cause significant reversion in the OPV recently^25^.

In this study, 2 microRNAs, viz. let-7a and miR-124a target genes, and the G64R (3D^pol^) mutation was engineered in the genome of the EV-A71 partial deletion mutant (∆11 bp in the 5′-NTR) to limit the degree of escape mutants. The let-7a and miR-124a target genes were incorporated into specific locations in the EV-A71 genome as these genetic regions were able to tolerate large insertions without any adverse effect on viral replication. The pIY vaccine strain carrying the miRNAs let-7a and miR-124a had decreased viral replication in both RD and SHYSY-5Y cells due to the presence of the corresponding cognate miRNAs in these two cell lines. The NTERA-2 cells did not express either miR-124a or let-7a. This was observed in growth kinetics study that the pIY strain from infected NTERA-2 cells yielded significantly higher RNA copy number than the pIY strain from infected SHYSY-5Y or RD cells at every hour post-infection. The reduction in RNA copy number of the pIY strain in RD and SHSY-5Y cells were confirmed to be due to the presence of the miRNA target genes incorporated in the viral genome as the addition of inhibitors to both let-7a and miR124a caused a de-repression of the specific targeted miRNA in the viral genome.

The efficacy of the MMS and pIY vaccine strains to elicit immune responses against EV-A71 and ameliorate severe infection in mice when challenged with the MAV were evaluated in the present study. When either of the vaccine strains was administered, the MMS or the pIY vaccine strain was observed to be effective in inhibiting EV-A71 replication in the murine model. This demonstrated that both the MMS and the pIY vaccine strains were able to prevent the development of hind limb paralysis in mice receiving the MMS or the pIY vaccine strain via the i.p. route. Mice splenocytes were also tested *ex vivo* against different vaccine strains for IFN-γ response. Interestingly, both the pIY vaccine strain and IV did not elicit levels of IFN-γ as high as the MMS strain. This could be due to the presence of the ubiquitous let-7a in splenocyte cells which was able to bind to the let-7a complementary sequence in the miRNA strain, hence reducing viral replication. This would in turn decrease the viral load but still retained a sufficient amount of immunogen needed to elicit a robust IFN-γ response. The expression of IFN-γ in particular, promotes cell-mediated immunity that is crucial for long-term memory and life-long protection from viral infections^26^.

As for the IV, the strain induced a poor T cell response as there is no viral multiplication in an IV and hence the reduced antigen levels could not sustain a prolonged antibody response. However, it has been reported that a formaldehyde-inactivated vaccine was able to protect 100% of immunized mice from weight loss, paralysis, and death. This could be due to the cross protective neutralizing antibodies (NtAbs) as the IV was able to neutralize sub-genotypes B3, B4 and C1 to C5, with the highest NtAbs against sub-genotypes B3 and B4 (≥ 1:512). The IV in this study was also able to neutralize sub-genotypes B3, B4 and C1 to C5 (1:32), but the lowest levels of NtAbs were against sub-genotypes B3 and B4 (1:16). The discrepancy could be attributed to different methods of preparing the IV, using formaldehyde instead of heat-inactivation and different mice strains (ICR versus BALB/c) were used.

Notably, the MAV strain utilized for challenge by Ong *et al*. (2010) was similar to the one used to challenge the mice in this study^27^. The constraint of this MAV strain is that it is only effective in eliciting hind limb paralysis and death in mice that were 2 weeks old or younger. This limited the study design to only incorporate 1 booster vaccination and a time lapse of only 1 week between inoculations. The immaturity of the immune system of such young mice could result in lower levels of IFN-γ expression, than would have been elicited in older mice. Hence, it would be desirable for further studies to utilize a different challenge strain that could produce paralysis symptoms in older mice. This is important that the mice could be re-challenged with the MAV strain at a later stage to assess the long-term protective efficacy of the vaccine strains and to measure the expression levels of IFN-γ. It would be interesting to note any changes in the levels of IFN-γ as this cytokine is a vital component in the cellular immune response. The vaccination studies could be evaluated with both vaccine strains using IFN-γ−/− mice to assess if the protective efficacy obtained in this study was indeed due to the IFN-γ expression. It is notable that the MMS and pIY vaccine strains were able to induce elevated IFN-γ responses, similar to the virus-like particle (VLP) vaccine strain constructed by Zhao *et al*. (2015). The ChiEV-A71 VLPs were able to elicit high NtAb titers of ≥ 1:64 and IFN-γ amounts of approximately 650 SFU/million splenocyte cells in immunized mice at 2-weeks after prime immunization. This was comparable to the levels of IFN-γ produced by the pIY vaccine strain at 670 SFU/million cells. The IV produced by Zhao *et al*. (2015) induced higher IFN-γ at 580 SFU/million cells, compared to the levels produced by the IV from this study at 380 SFU/million cells^28^. This could be due to the different age or breed of mice and different dosages of the vaccine strains (10 µg EV-A71 VLPs vs 10^4^ PFU/ml vaccine).

The development of successful vaccines would require the production of strong and robust IFN-γ responses that play a vital role in the antiviral defense against EV-A71 infection. Mice deficient in IFN-γ receptors had higher fatality rates and more severe disease progression with EV-A71 infection. This was attributed to the role of viral proteins 2A and 3D that inhibited IFN regulatory factor 1 (IRF1) transactivation, prior to a loss of STAT1 nuclear translocation. Blocking the 2A or 3D activity was able to restore IFN-γ signaling, whilst neuro-2a cells pre-treated with IFN-γ had decreased viral yield^29^. IFN-γ has been found to have antiviral functions and can decrease viral load in tissues of EV-A71 infected mice^30,31^. These activities are vital for viral clearance and might explain how the MMS, pIY vaccine strain and the IV were able to confer 100% protection against hind limb paralysis in vaccinated mice. The vaccination would have induced the production of antigen presenting cells, natural killer cells, and T cells that elicited the antiviral IFN-γ which could effectively kill virus-infected cells.

High levels of IFN-γ have been shown to mediate viral clearance in lymphoid tissues and increase the survival rate of mice. AG129 mice lacking Type I and II Interferon receptors demonstrated progressive limb paralysis prior to death and histopathological examinations revealed a tremendous amount of damage in the brainstem and anterior horn areas^32^. In a separate study, a mouse adapted EV-A71 strain produced 100% lethality in 10-week old AG129 mice. The strain was only able to cause 17% lethality in A129 mice that lacked only Type 1 (α/β) Interferon receptors, indicative that adult AG129 mice are vulnerable to infection by MAV in the absence of a fully functional IFN response^33^. In addition, Shen *et al*. (2013) discovered that infection with EV-A71 increased the levels of interferon-gamma-inducible protein-10 (IP-10), which in turn increased the expression of IFN-γ and the infiltration of CD8^+^ T cells to aid survival of mice^34^. This was similarly observed for the West Nile virus (WNV) as a lack of IFN-γ signaling demonstrated higher mortality to lethal infection with an increase in death from 30% (WT mice) to 90% in IFN-γ−/− or IFN-γR−/− mice. The protective role of IFN-γ against WNV was antiviral and this happened in peripheral lymphoid tissues, preventing its spread to the CNS^35^. It was shown that IFN-γ was critical for hepatitis viral clearance from the CNS oligodendroglia. Mice deficient in IFN-γ secretion (IFN-gamma^0/0^) demonstrated an inability to control viral replication through increased clinical symptoms and lethality linked with persistent virus^36^. It was also observed for dengue virus (DENV), presence of IFN-γ prevented systemic infection and paralysis in IFN-α/β receptor-deficient mice. In these doubly-deficient AG129 mice, IFN-γ restricted DENV replication in the spleen and bone marrow within 24 h of infection. This was attributed to a 140-fold greater resistance against systemic vascular leakage commonly associated with dengue infection^37^. Nevertheless, current knowledge of human cellular immunity towards EV-A71 remain limited. To date, only several studies have investigated the role of cellular immunity against EV-A71 in small groups of human subjects based on *in silico* predicted peptides^38,39^. Hence, the role of T cells in EV-A71 protection remains to be explored.

In this study, both the MMS and pIY strain were able to elicit high IFN-γ levels (651–753 SFU/million splenocytes) that may have conferred protection against hind limb paralysis in the 4-week old mice. A West Nile vaccine construct (WNVΔVax) designed by Brostoff *et al*.^40^ had increased safety and stability by similarly using miRNA targets with perfect complementarity for miR-124a and by incorporating multiple target sequences in cassettes with sufficient spacing for multiplicative silencing. In addition, viral replication was decreased in neuronal cells which expressed cognate miR-124a, as it was vital for the prevention of fatal brain encephalitis and meningitis. This attenuation provides an additional level of safety, and decreased the potential for selection of mutants in non-neuronal cells. At the same time, there was viral replication in peripheral cells (non-neuronal cells) to induce strong NtAb titers (PRNT_90_ ≥ 500–1700). Interestingly, all the detectable NtAb titers, regardless of magnitude, was found to be protective against challenge studies in CD-1 mice^40^.

As the pIY vaccine strain induced lower immunogenicity than the MMS and IV, this could be further increased by incorporating miR-155 into the strain. The insertion of miR-155 in the influenza A (H3N2) viral genome was found to increase immunogenicity in 8-week old mice. The C57.BL/6 mice vaccinated with the X31–155 LAV elicited significantly higher levels of NtAb titres (1:256)^41^. A major concern with miRNA-based LAVs would be its genetic instability, and hence the possibility of escape mutants arising. Barnes *et al*.^12^ discovered that the recombinant poliovirus carrying miRNA let-7a did not replicate well in most tissues. This would then elicit a weaker immune response and served as a vaccine with low immunogenic properties. Although the poliovirus carrying the miR-124a (PV-124) could not replicate in neuronal tissues, it was able to grow extensively in other tissues. The virus was found to accumulate escape mutations that could prevent degradation of the viral genome. This also increases the potential for the PV-124 to revert to the WT genotype. In fact, several mice had low titers of mutated PV-124 virus in their spinal cords. Hence, to prevent viral escape from miRNA-mediated interference in replication, it is desirable to incorporate two different miRNA target sequences into the viral genome or by inserting the sequences in locations flanking regions of the 3′NTR that is efficient for viral replication.

To address any genetic stability or reversion to virulence, genetic stability studies of both the pIY miRNA strain and the MMS were evaluated *in vitro*. In the pIY vaccine strain, the 2 miRNA target genes, partial deletion (∆11 bp) and the G64R (3D^pol^) mutations were stable and did not revert to WT even after 20 serial passages in RD cells. There was no single nucleotide substitution or loss of the miRNA target sequences after these passages. Since no escape mutants were observed for both miRNA targets, the miRNA vaccine strain of EV-A71 (pIY) is deemed to be genetically stable. In the MMS strain, each of the 6 single site mutations and the deletion mutation were stable and did not revert to the WT genotype after 20 passages. Future directions could look into *in vivo* serial passaging in mice to assess any potential of reversion to the WT. If there is no escape from miRNA targeting through point mutations, this further validates the safety of the strategy. Other neuronal-specific miRNA target genes such as miR-129, miR-154 or miR-433 could be incorporated into the miRNA-based EV-71 vaccine strain, in addition to miR-124a and let-7a as described in this study. Heterologous miRNAs could reduce selection pressure as reported for miR-124a^42^.

The absence of US FDA-approved vaccines for prevention of fatal HFMD caused by EV-A71 has intensified research into EV-A71 vaccine development. The IV is the lead vaccine candidate to enter the China market but it has only 80% efficacy against severe HFMD due to the poor development of the immune system in children. Therefore, there is a need to develop alternative vaccines such as the LAV that could induce long term cellular and humoral immune responses. Both the MMS and the miRNA-based pIY vaccine strains are promising vaccine candidates against severe HFMD as it conferred 100% protection against paralysis and elicited high levels of protective IFN-γ *ex vivo*. An absence of EV-A71 viral antigens in the skeletal muscles and spinal cords of mice vaccinated with both LAVs indicated the lack of infection in these sites. Hence, the MMS and the pIY vaccine strains are ready to be evaluated in larger animals such as the cynomolgus monkeys.

## Materials and Methods

### Ethics statement

All animal experimentations were performed in accordance with the Guidelines for Animal Experiments of the National Institute of Infectious Diseases (NIID, Tokyo, Japan). Experimental procedures were reviewed and approved by the Institutional Animal Care and Use Committee, University of Malaya, Malaysia (IACUC Approval Number: 2016-191103/SUN/R/IYPT) and Sunway University Research Ethics Committee (IACUC Approval Number: SUREC 2016/010). Mice were housed in individually ventilated cages and provided with water and standard lab rodent diet. The mice were monitored daily for health and clinical signs. More than 20% body weight loss, hunching and ruffled fur were used as the criteria for euthanasia by CO_2_ inhalation.

### Cells and viruses

Human rhabdomyosarcoma (RD; ATCC #CCL-136), neuroblastoma (SH-SY5Y; ATCC #CRL-2266), African green monkey kidney cells (Vero; ATCC # CCL-81) and embryonal carcinoma cells (NTERA-2; ATCC # HTB-106) cells were maintained in DMEM (Invitrogen, USA) that was supplemented with 10% FBS (Gibco, Calif., USA), 1% L-glutamine, 1% non-essential amino acids and 1% penicillin/streptomycin. A neuro-virulent EV-A71 strain 5865/SIN/00009 (Accession No.: AF316321; sub-genotype B4; designated as Strain 41) was isolated from a deceased child during the Singapore HFMD outbreak in October 2000.

### Insertion of miRNA target sequences into the EV-A71 genome

Specific primers were designed for the insertion of target sequences complementary to the two miRNAs (let-7a and miR124a) using Overlapping Extension PCR into two locations within the EV-A71 genome that contained the partial deletion (∆11 bp) and G64R mutation (Table 2). Fragment 1 (768 bp) consisted of the 5′-NTR and let-7a target sequence (AACTATACAACCTACTACCTCA). Fragment 2 (2608 bp) comprised all the EV-A71 structural genes (VP1-VP4) flanked by the target sequences of let-7a and miR-124a (TGGCATTCACCGCGTGCCTTA). Fragment 3 (4099 bp) covered the entire non-structural genes (2A-2C and 3A-3D) alongside with the miR-124a target sequence.

**Table 2:** Nucleotide sequence of primers designed for the insertion of miRNA target sequences into EV-A71 genome.

Primer Designation	Nucleotide sequences
F1 (Fwd 5′-NTR)	5′-TTAAAACAGCTGTGGGTTGTACCCAC-3′
R1 (Rev let-7a)	5′-**TGAGGTAGTAGGTTGTATAGTT**GTTTGATTGTATTGAGG-3′
F2 (Fwd let 7a)	5′-**AACTATACAACCTACTACCTCA**ATGGGCTCACAGGTGTC-3′
F2: (Fwd let 7a**-scr**)	5′-**AATTACACGACTTATTATTTGA**ATGGGCTCACAGGTGTC-3′
R2: (let 7a**-scr**):	5′-**TATGGGACCCGCTGTATCCCT**AAGGGTAGTAATGGCAG-3′
R2 (Rev miR124a)	5′-**TAAGGCACGCGGTGAATGCCA**AAGGGTAGTAATGGCAG-3′
F3 (Fwd miR-124a)	5′-**TGGCATTCACCGCGTGCCTTA**GGAAAGTTCGGCCAGC-3′
F3 (Fwd miR-124a**-scr**)	5′-**TAGCGTTAACTGCATGGCGCA**GGAAAGTTCGGCCAGC- 3′
R3 (Rev 3′-NTR)	5′-GCTATTCCGGTTATAACAAATTTACCCCCACTAG-3′

Nucleotides that are in bold refer to the two miRNA target sequences. Primers designated let-7a-scr or miR124a-scr were primers designed to incorporate scrambled nucleotides into target sequences to produce virus controls that had disrupted base pairing with endogenous miRNA whilst conserving the WT encoded amino acids.

Then, the three fragments (F1 + F2 and F3) were ligated using Overlapping Extension PCR. The PCR was performed with the Phusion Hot Start II DNA Polymerase (2 U/µL) (Thermo Scientific, Calif., USA) and the 2-step cycling parameters which included an initial denaturation for 30 s at 98 °C, and 30 cycles of denaturation at 98 °C for 10 s, annealing at 72 °C for 30 s/kb, followed by a final extension at 72 °C for 10 minutes.

### Cloning of EV-A71 carrying microRNA target sequences into the pCR-XL-TOPO vector

The  7.5 kb EV-A71 genome was cloned into the 3.5 kb pCR-XL-TOPO vector according to the manufacturer’s instructions (Invitrogen, Calif., USA). The recombinant pTOPO-miR7124 plasmid was subjected to DNA sequencing of the selected modified regions to confirm the presence of the microRNA target genes. The digestion and phenol chloroform purification of the recombinant plasmid DNA was described previously^43^.

### Reverse transcription of EV-A71 RNA into cDNA

The EV-A71 genomic RNA was reverse transcribed into cDNA using SuperScript III First-Strand Synthesis System (Invitrogen, Calif., USA) according to the manufacturer’s instructions. PCR amplification of the cDNA was performed with the DreamTaq DNA polymerase (Thermo Scientific, Calif., USA) and the 3-step cycling parameters which included an initial denaturation for 2 minutes at 98 °C, 25 cycles of denaturation at 98 °C for 10 s, 60 °C for 20 s and 72 °C for 4 minutes.

### *In vitro* transcription of DNA into infectious RNA

RNA transcription from cDNA was carried out using RiboMAX™ Large Scale RNA Production System-SP6 (Promega, Calif., USA). The reaction was set up in 20 µl of reaction volume following the manufacturer’s protocol. The reaction mixture was subjected to incubation at 37 °C for 4 h, followed by DNase treatment for 30 minutes. The DNase (Promega, Calif., USA) was added to the *in vitro* transcription reaction at a final concentration of 1 U per µg of DNA template. The *in vitro* transcribed EV-A71 RNA was visualized by agarose gel electrophoresis (90 V for 90 min) prior to its use in transfection of RD cells.

### Transfection of infectious viral RNA

Overnight grown RD/SHSY-5Y/NTERA-2 cells (1.5 × 10^5^ cells/well in a 24-well plate) were prepared and used for transfection. *In vitro* transcribed viral RNA (1 μg) was transfected into each well containing cells. The RNA in Opti-MEM was mixed with the Lipofectamine 2000 reagent (Invitrogen, Calif., USA) in Opti-MEM and incubated at room temperature for 20 minutes. Thereafter, the RNA-Lipofectamine mixture was added to the cells drop-by-drop. Four hours after transfection, the transfection medium was removed and replaced with 500 μL 10% FBS DMEM without penicillin/streptomycin (Gibco, Massachusetts, USA).

### Addition of anti-miR inhibitors to different cell lines

Custom miRCURY LNA miRNA Power inhibitors and scrambled sequence inhibitors for let-7a and miR-124a were purchased from Qiagen (Calif., USA). The gene sequences for the anti-miR inhibitors for let-7a and miR-124a are AACUAUACAACCUACUACCUCA and UGGCAUUCACCGCGUGCCUUA, respectively. The scrambled gene sequences for the anti-miR inhibitors for let-7a and miR-124a are AAAUAGACCACAUAAUAACUAA and UGUCAUGCAACGAGUUCCGUA, respectively. In both cases, 1.5 × 10^5^ RD/SHSY-5Y/NTERA-2 cells were seeded in wells of a 24-well plate and transfected with 25 pmol anti-miR inhibitor specific for let-7a and 25 pmol anti-miR inhibitor specific for miR-124a with Lipofectamine 2000 reagent (Invitrogen, Calif., USA). After 24 h incubation, viral RNA transfection was carried out. Subsequently after 4 h, the transfection medium was removed and replaced with 500 μl 10% FBS DMEM without penicillin/streptomycin (Gibco, Massachusetts, USA).

### Plaque assay

The amount of viral titer was quantified by the plaque assay as described previously^16^.

### Tissue culture infectious dose (TCID_50_) assay

TCID_50_ is defined as the quantity of virus that will produce a cytopathic effect in 50% of the cultures inoculated. RD cells (3.0 × 10^4^ cells/well) were seeded in a 96-well plate a day before. On the next day, 10-fold serial dilutions of virus suspension were made using DMEM serum-free media as a diluent. This was carried out in quadruplicates in 96-well plates. The negative control wells contained uninfected RD cells. The plates were incubated at 37 °C and observed for CPE for up to 48 h. The TCID_50_/ml value was determined in RD cells using the Reed and Muench formula as described below:

$$\begin{array}{l}{{\rm{TCID}}}_{50}/\text{ml}=\frac{( \% \,{\rm{wells}}\,{\rm{infected}}\,{\rm{at}}\,{\rm{dilution}}\,{\rm{next}}\,{\rm{above}}\,50 \% )-(50 \% )}{( \% \,{\rm{wells}}\,{\rm{infected}}\,{\rm{at}}\,{\rm{dilution}}\,{\rm{next}}\,{\rm{above}}\,50 \% )\,-\,( \% \,{\rm{wells}}\,{\rm{infected}}\,{\rm{at}}\,{\rm{dilution}}\,{\rm{next}}\,{\rm{below}}\,50 \% )}\\ {\bf{Whereby}}\,{\bf{h}}={\rm{dilution}}\,{\rm{factor}}\end{array}$$

### RNA extraction from transfected cells

Viral RNA extraction was carried out from the supernatant of the transfected cells after 80% CPE was observed using the QIAamp® Viral RNA Mini Kit (Qiagen, Calif., USA) as previously described^16^.

### Real time Reverse Transcriptase Polymerase Chain Reaction (RT-PCR)

Real-time Reverse Transcriptase Polymerase Chain Reaction (RT-PCR) was carried out utilizing the Touch^TM^ Real-Time Reverse Transcriptase PCR Detection System, CFX96 (Bio-Rad, Calif., USA). The real-time reverse transcriptase PCR was performed with the TaqMan Fast Virus 1-Step Master Mix (Applied Biosystems, Calif., USA) utilizing the primers and the TaqMan probe designed for the quantitation of viral RNA copy number determination.

### Genetic stability of the MMS and pIY vaccine strains

Genetic stability of the mutations in the MMS and the two miRNA target sequences in the pIY vaccine strain was examined during serial passages of the respective vaccine strains in RD cell cultures. RD cells were seeded with 500 μl of DMEM containing 5% FBS and 3.0 × 10^5^ cell per well in a 24-well plate. The growth medium was subsequently removed and 150 μl of supernatant containing each vaccine strain was added per well. The plate was incubated at 37 °C in a 5% CO_2_ incubator for 1 h. The plate was frozen and thawed three times and the contents of the well were centrifuged to remove cell debris. An aliquot of 150 μl of the cleared supernatant was used for the next passage of the MMS and pIY vaccine strains in triplicates for 20 passages. Stability of each of the mutations in the MMS or the miRNA target genes introduced into the miRNA vaccine strain was assessed through sequencing.

### EV-A71 mice challenge studies

Groups of 2-week old mice (n = 5) were intraperitoneally (i.p.) immunized with Freund’s complete adjuvant containing either 0.1 ml EV-A71 MMS (10^4^ PFU/ml), pIY vaccine strain (10^4^ PFU/ml) or the heat-inactivated (56 °C, 30 min) EV-A71 strain 41 (IV) (10^4^ PFU/ml) using a 26 G needle. The control mice (n = 5) did not receive any inoculation of the vaccine strains, but was given 0.1 mL of PBS instead. After 1 week, mice were given the first booster dose of each vaccine strain (10^4^ PFU/ml each) in Freund’s incomplete adjuvant or PBS. After 14 days from the first immunization, all mice groups were challenged with the mouse adapted virus (MAV) by the i.p. route with 10^5^ CCID_50_/ml. Clinical scores were defined as follows: 0, healthy; 1, ruffled hair, hunchbacked; 2, reduced mobility; 3, limb weakness; 4, hind limb paralysis; and 5, moribund and death. At the end of the study (day 24), the mice were euthanized using CO_2_ asphyxiation. The skeletal muscles and spinal cords were subjected to immunohistochemistry analysis. The spleens were harvested for the Enzyme-Linked ImmunoSpot (ELISPOT) assay.

### Preparation of murine splenocytes

The murine splenocytes were prepared according to a standard protocol^44^.

### Mice *ex vivo* IFN-γ ELISPOT assay

Splenocyte cells were re-suspended to a concentration of 2.5 × 10^6^/ml in complete RPMI and seeded at a density of 2.5 × 10^5^ per well in a 96 well ELISPOT plate that contained immobilized IFN-γ-specific monoclonal antibodies (mAb) from the IFN-γ ELISPOT kit (Mabtech, Ohio, USA). Wells that contained 5 µg/ml of purified phytohemagglutinin-M (PHA-M) (Sigma Aldrich, Calif., USA) served as positive control wells. The MMS and pIY vaccine strains or the IV, combined with Freund’s incomplete adjuvant were each added to the wells at a final concentration of 10^4^ PFU/ml for overnight stimulation. After 24 h, the cells were removed and the detection mAb which was biotinylated was added. Thereafter, a streptavidin-enzyme conjugate was added and after addition of 3,3′,5,5′-Tetramethylbenzidine (TMB) substrate, the number of spots were counted using the automated ELISPOT reader (Cellular Technology Limited, Ohio, USA) to determine the frequency of IFN-γ secreting cells.

### Immunohistochemical analysis

On day 10-post infection, 20 sets of skeletal muscles and spinal cords of the ICR mice inoculated with either the MMS or pIY vaccine strains as well as the IV and PBS-injected mice were harvested and fixed in 10% buffered formalin for 1 week. Cryosections of 4 μm from the formalin-fixed tissues were made and mounted on Poly-L-lysine microscopic slides. Any endogenous peroxidase activity was quenched by incubating the tissue cryosections with an endogenous peroxidase blocking reagent for 20 min. The tissues were then incubated with murine monoclonal antibody against EV-A71 (Merck, New Jersey, USA). This was followed by incubation with a peroxidase labelled polymer conjugated to goat anti-mouse IgG for 30 min. A brownish-red colored peroxidase stain was developed using 3,3′-diaminobenzidine (DAB) substrate with a 5 min incubation and counterstained with hematoxylin (Sigma Aldrich, Calif., USA).

### Statistical analysis

All the results were expressed as mean ± standard deviations (indicated by error bars in graph). Statistical analysis was performed by the two-tailed unpaired t-test or one-way ANOVA using GraphPad Prism 7.0 for Windows (GraphPad Software, Inc., San Diego, USA). All data not following a normal distribution were tested for statistical significance by a Mann–Whitney *U*-test; *P* values of less than 0.05 were considered as statistically significant.

## Supplementary information

Raw Data

## Data Availability

The raw data required to reproduce these findings are available.
