# Supplementary figures and images for: Development of live attenuated Enterovirus 71 vaccine strains that confer protection against lethal challenge in mice
Source: Sci Rep. 2019 Mar 18;9:4805. doi: 10.1038/s41598-019-41285-z (PMC6423319; doi:10.1038/s41598-019-41285-z)

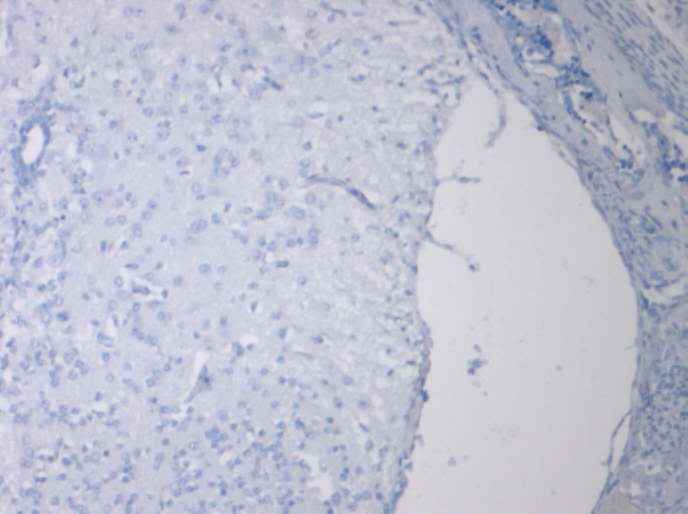

Supplement: Supplementary file 1 — Raw Data [file 41598_2019_41285_MOESM1_ESM.zip › Fig 7_IHC Data/Healthy Spinal cord IHC ENV 1IN100_10X.jpg]

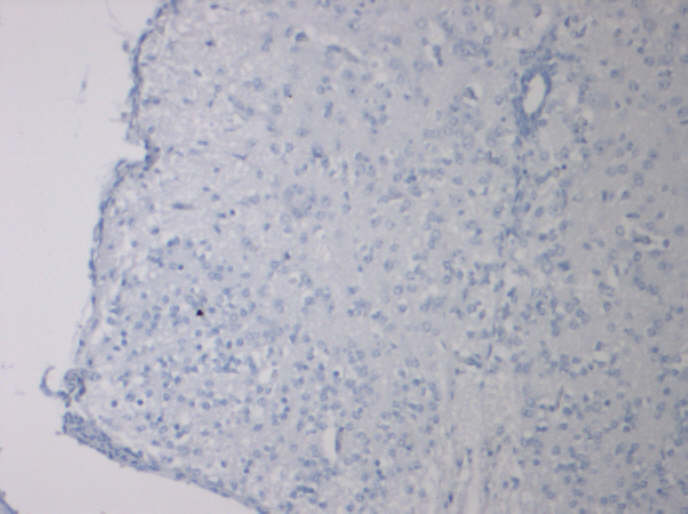

Supplement: Supplementary file 1 — Raw Data [file 41598_2019_41285_MOESM1_ESM.zip › Fig 7_IHC Data/Healthy Spinal cord pIY_40X.jpg]

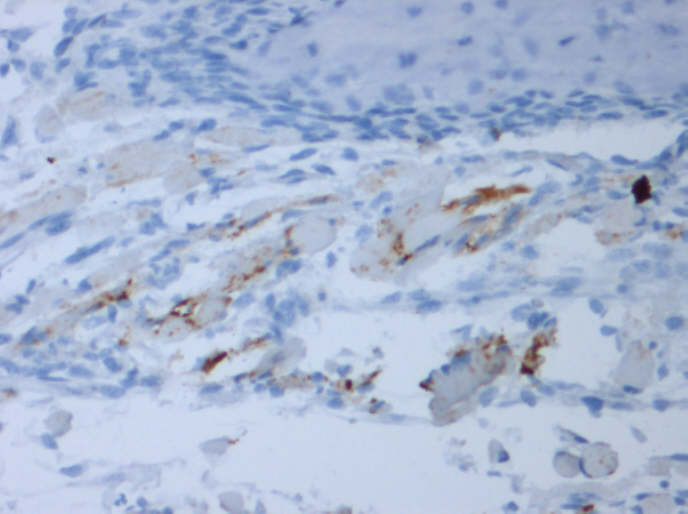

Supplement: Supplementary file 1 — Raw Data [file 41598_2019_41285_MOESM1_ESM.zip › Fig 7_IHC Data/Skeletal muscle IHC ENV 1IN100_20X[1].jpg]

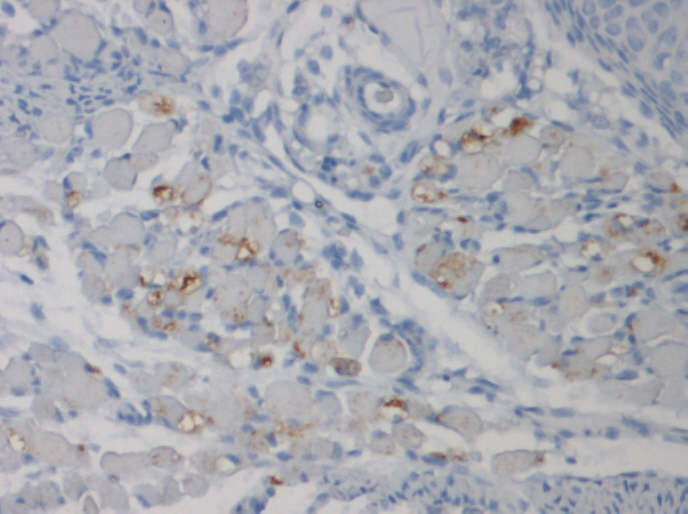

Supplement: Supplementary file 1 — Raw Data [file 41598_2019_41285_MOESM1_ESM.zip › Fig 7_IHC Data/Skeletal muscle IHC ENV 1IN100_20X[2].jpg]

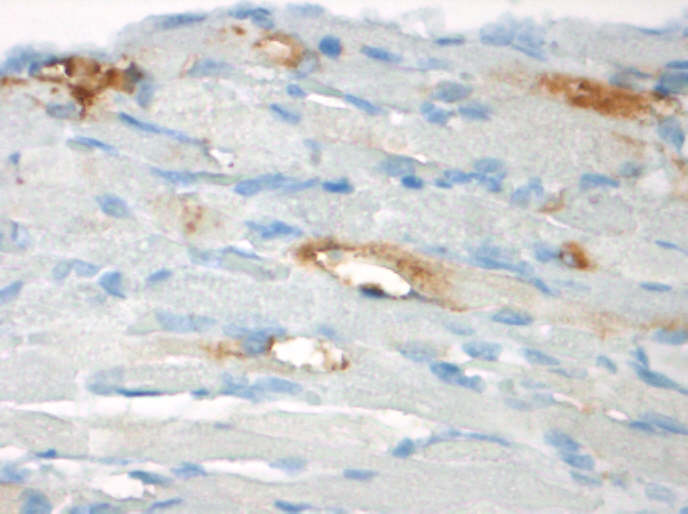

Supplement: Supplementary file 1 — Raw Data [file 41598_2019_41285_MOESM1_ESM.zip › Fig 7_IHC Data/Skeletal muscle X40.jpg]

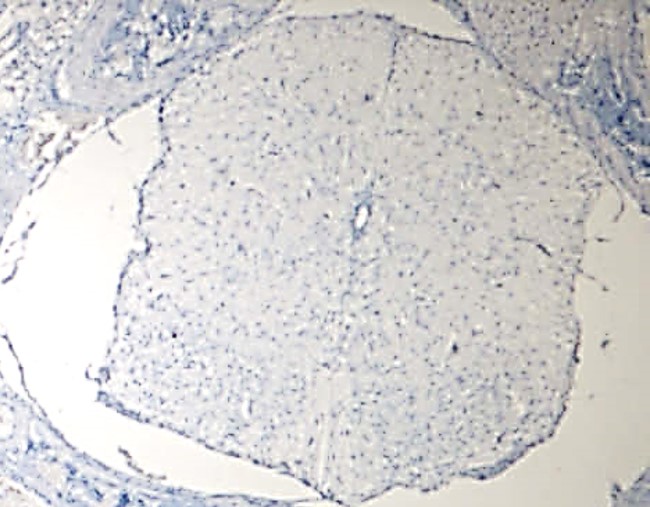

Supplement: Supplementary file 1 — Raw Data [file 41598_2019_41285_MOESM1_ESM.zip › Fig 7_IHC Data/Spinal cord muscle IHC ENV 1IN100_4X.jpg]

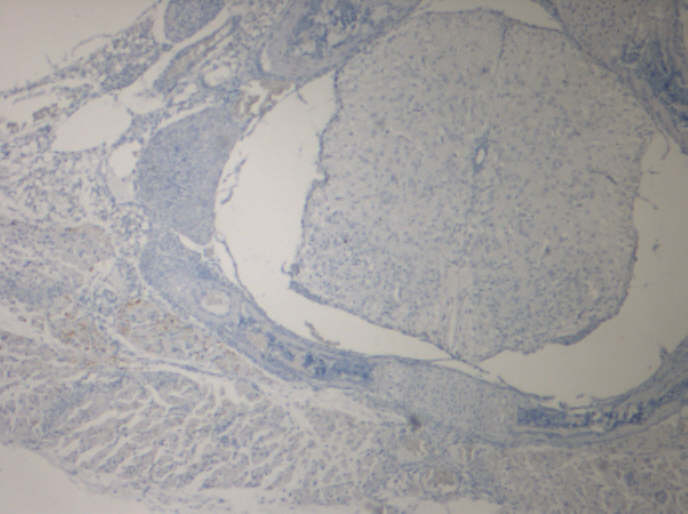

Supplement: Supplementary file 1 — Raw Data [file 41598_2019_41285_MOESM1_ESM.zip › Fig 7_IHC Data/Spinal cord PBS_10X.jpg]
